# Predictive and prognostic significance of telomerase levels/telomere length in tissues and peripheral blood in head and neck squamous cell carcinoma

**DOI:** 10.1038/s41598-019-54028-x

**Published:** 2019-11-26

**Authors:** Paolo Boscolo-Rizzo, Enrica Rampazzo, Jerry Polesel, Silvia Giunco, Anna Menegaldo, Monica Mantovani, Marco Stellin, Luigia Bandolin, Giacomo Spinato, Annarosa Del Mistro, Daniele Borsetto, Jonathan Fussey, Giancarlo Tirelli, Maria Cristina Da Mosto, Anita De Rossi

**Affiliations:** 1 Department of Neurosciences, Section of Otolaryngology and Regional Centre for Head and Neck Cancer, University of Padova, Treviso, Italy; 2 Department of Surgery, Oncology and Gastroenterology, Section of Oncology and Immunology, University of Padova, Padova, Italy; 3 Unit of Cancer Epidemiology, Centro di Riferimento Oncologico di Aviano (CRO) IRCCS, Aviano, Italy; 4 Immunology and Molecular Oncology Unit, Istituto Oncologico Veneto IOV-IRCCS, Padova, Italy; 5 Department of ENT, Addenbrooke’s Hospital, Cambridge University Hospitals, Cambridge, UK; 6 Department of Otolaryngology, Royal Devon and Exeter Hospital, Exeter, UK; 7 Head and Neck Department, Hospital of Cattinara, University of Trieste, Trieste, Italy

**Keywords:** Head and neck cancer, Head and neck cancer

## Abstract

A growing body of evidence indicates that the expression of TERT, the catalytic subunit of telomerase, is a biological marker of progression in several cancers. We investigated the predictive and prognostic role of TERT levels and telomere length in tissues and peripheral blood in patients with head and neck squamous cell carcinoma (HNSCC). High *TERT* levels in cancer tissues were independently associated with worse response to therapy (odds ratio [OR]:6.26), regional failure (hazard ratio [HR]:5.75), progression (HR:2.12), and death (HR:3.53). Longer telomeres in the mucosa surrounding the tumor (SM) were independently associated with a lower risk of mucosal failure (HR:0.39). While telomere length in peripheral blood mononuclear cells (PBMC) significantly decreased with age, no correlation was found between age and telomere length in SM. No associations were found between *TERT* levels in plasma and telomere length in PBMC and the prognostic variables. High levels of *TERT* transcripts in cancer cells represent a reliable prognostic marker for identifying HNSCC patients with risk of progression. The altered relationship of telomere length to age in SM compared with PBMC suggests that in a subset of cases the phenotypically normal SM constitutes an acquired telomere-shortened epithelial field prone to genetic instability.

## Introduction

Head and neck squamous cell carcinomas (HNSCCs) are a group of neoplasms developing in epithelia lining the upper aero-digestive tract (UADT, i.e. the oral cavity, the oropharynx, the hypopharynx, and the larynx). HNSCCs are caused predominantly by chemically (through tobacco and/or alcohol exposure) or virally induced carcinogenesis after infection by human papillomavirus (HPV)^1^. Tobacco- and alcohol-related HNSCC and HPV-driven HNSCC are two biologically and clinically distinct entities, with the latter being associated with low mutational burden and significantly longer overall survival^2^. While HPV-driven HNSCCs show wild-type *TP53* sequences and the presence of viral oncogenes E6 and E7, HPV-negative disease more commonly harbors *TP53* mutations and chromosomal instability. However, about 20% of HPV-negative HNSCCs harbor wild-type *TP53* sequences, low numbers of genetic mutations and low chromosomal instability, thus representing a subgroup of genetically distinct HPV-negative disease^3^.

Moreover, next-generation sequencing techniques have revealed the intricate genetic makeup of HNSCC^4^. Both HPV-driven and non-HPV-driven tumors can indeed be subclassified by gene expression profiling, and different sets of mutations have been observed among tumors from different individuals with the same type of cancer. Additionally, recent studies have demonstrated significant intratumoral heterogeneity whereby a single tumor contains a diverse collection of cells harboring distinct molecular signatures^5^. Thus, despite possessing similar histological characteristics, HNSCC is a tremendously heterogeneous disease.

To date, there are no robust molecular markers useful in planning management for patients with HNSCC and, despite their intrinsic heterogeneity, all HNSCCs are similarly treated^1^. Consequently, even within an individual subsite, the clinical course of HNSCC, including response to therapy, disease progression and metastasis, varies widely with outcome being poorly predicted by clinical or histological features.

Although numerous genetic alterations involving a variety of different pathways contribute to the development and progression of HNSCC, 85% of cancers acquire the capability to replicate indefinitely through the re-activation of telomerase, a ribonucleoprotein complex containing an internal RNA component and a catalytic protein, TERT, with telomere specific reverse transcriptase activity^6^.

Despite somewhat divergent conclusions^7,8^, a substantial body of studies have uncovered novel extra-telomeric non-canonical functions of telomerase, many of which affect cellular processes including signaling pathways for the regulation of cell survival, resistance to stress, and apoptosis^9^. Importantly, through the interaction of TERT with the Wnt/β-catenin and the NF-kB signaling pathways, the expression of telomerase may affect cancer invasiveness and metastasis^10–12^. Thus, levels of telomerase in cancer cells may influence the response to treatment as well as cancer progression and metastasis, making it a potentially attractive predictive and prognostic marker. Prior to telomerase activation, telomere dynamics plays an essential role in the early stage of carcinogenesis. Telomere attrition appears indeed to be a driver of genetic instability and short telomeres are consistently found in HNSCC precursors and in mucosa adjacent to pre-neoplastic areas and invasive cancer^13,14^.

Moreover, mechanistic studies have clearly demonstrated that mammalian cells lacking telomerase activity show progressive telomere shortening that, in late generation mice, results in end-end chromosome fusions and deletions upon irradiation^15,16^. Thus, high levels of TERT, by stabilizing longer telomeres may indirectly confer cancer cells with an increased resistance to radiotherapy and contribute to tumor progression.

Our previous research has demonstrated high *TERT* levels in cancer tissues to be associated with progression at regional and distant sites and higher risk of death, and short telomeres in the mucosa surrounding the tumor (SM) to predict a higher risk of mucosal failure^13^. These observations have raised several questions: are shortened telomeres in SM the consequence of greater cell proliferation or are they linked to an individual’s constitutive telomere length? What is the significance of telomere length in peripheral blood mononuclear cells (PBMC)? What is the significance of cell-free circulating plasma *TERT* mRNA? To answer these questions, in this new prospective study, we also collected blood samples in addition to cancer tissue and normal SM. We estimated telomere length and *TERT* levels and their relationship in cancer tissue, SM, and peripheral blood, and we investigated their predictive and prognostic roles in patients with HNSCC.

## Results

### Demographic and clinical characteristics of patients

The clinical characteristics of the 101 patients are summarized in Supplementary Table 1. The study group had a median age of 65 years (range, 28–87 years) at presentation and included 74 (73%) male and 27 (27%) female patients. The female prevalence was significantly lower among tumors developing from the hypopharynx/larynx (*P* = 0.003; Supplementary Table 1). The majority of patients were ever smokers (73%) and ever drinkers (59%). However, ever drinking was less frequent in patients with oropharyngeal SCC (*P* = 0.018; Supplementary Table 1), which overall were more commonly diagnosed in advanced stage (*P* = 0.003; Supplementary Table 1). Six percent (n = 4/66) of patients submitted to primary surgical treatment had positive/close margins. Among the 66 patients treated with upfront surgery, 28 (42%) received adjuvant (chemo)radiotherapy.

### Telomere length in tumor, SM, and PBMC

**Figure 1:**
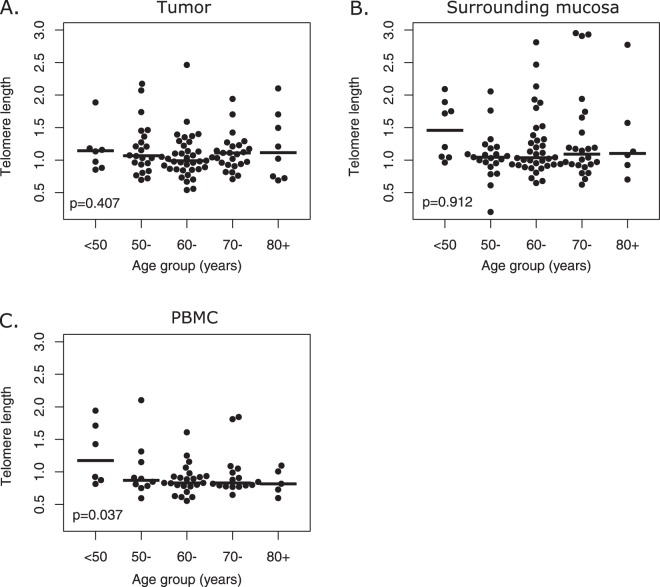
Distribution and median values of telomere length in tumor (**A**), surrounding mucosa (**B**) and peripheral blood mononuclear cells (PBMC) (**C**) according to age. Trend in values was tested through Analysis of Variance (ANOVA) with contrasts for linear trend.

Median telomere length in tumor cells, SM, and PBMC according to descriptive characteristics are reported in Table 1. The telomere length in tumor cells and in PBMC was not significantly associated with any of the descriptive variables investigated herein. On the other hand, telomere length in SM was significantly shorter in male patients (*P* = 0.032) and in ever drinkers (*P* = 0.004). No correlation was found between age and telomere length (Table 1). However, a finer classification of age revealed that telomere length in PBMC, but not in tumor and SM (Fig. 1A,B), significantly decreased with age (*P* = 0.037; Fig. 1C); no correlation was found between telomere length in SM and PBMC (r-Spearman = 0.22; *P* = 0.107). Additionally, no correlation existed between telomere length in SM and in the tumor, nor between telomere length in PBMC and in the tumor.

**Table 1:** Distribution of 101 patients with head and neck cancer and median values of telomere length and telomerase reverse transcriptase (TERT) level according to patient and tumor characteristics.

	n	(%)	Telomere length			TERT level		
			Tumor(n = 101)	SM(n = 96)	PBMC(n = 61)	Tumor(n = 93)	SM(n = 88)	Plasma(n = 94)
Gender								
Male	74	(73.3)	1.05	1.04	0.88	1128	427	32
Female	27	(26.7)	1.04	1.17	0.83	1946	437	0
Kruskall-Wallis test			p = 0.892	p = 0.032	p = 0.856	p = 0.023	p = 0.993	p = 0.175
Age (years)								
<60	30	(29.7)	1.10	1.06	0.90	1725	454	42
60–69	37	(36.6)	0.99	1.04	0.83	1356	401	37
≥70	34	(33.7)	1.11	1.10	0.82	779	446	0
Kruskall-Wallis test			p = 0.301	p = 0.970	p = 0.260	p = 0.116	p = 0.907	p = 0.654
Smoking habits								
Never	27	(26.7)	1.11	1.14	0.81	1524	349	27
Ever	74	(73.3)	1.04	1.04	0.89	1228	465	29
Kruskall-Wallis test			p = 0.332	p = 0.341	p = 0.308	p = 0.510	p = 0.338	p = 0.799
Drinking habits								
Never	41	(40.6)	1.09	1.17	0.87	1425	410	31
Ever	60	(59.4)	1.03	1.01	0.88	1269	465	17
Kruskall-Wallis test			p = 0.645	p = 0.004	p = 0.721	p = 0.821	p = 0.841	p = 0.759
Cancer site								
Oral cavity	27	(26.7)	1.15	1.16	1.08	1090	338	37
Oropharynx	22	(21.8)	1.06	1.08	0.88	1679	404	24
Hypopharynx/Larynx	52	(51.5)	0.99	1.03	0.85	964	527	23
Kruskall-Wallis test			p = 0.095	p = 0.296	p = 0.479	p = 0.108	p = 0.013	p = 0.912
cT								
T1-T2	52	(51.5)	1.07	1.13	0.84	739	384	30
T3-T4	49	(48.5)	1.04	1.04	0.89	1559	648	17
Kruskall-Wallis test			p = 0.510	p = 0.168	p = 0.119	p = 0.010	p = 0.094	p = 0.392
cN								
Negative	50	(49.5)	1.07	1.03	0.84	384	359	50
Positive	51	(50.5)	1.03	1.09	0.89	1679	632	14
Kruskall-Wallis test			p = 0.825	p = 0.193	p = 0.214	p < 0.001	p = 0.056	p = 0.351
Stage								
I-II	36	(35.6)	1.07	1.04	0.85	346	297	49
III-IV	65	(64.4)	1.04	1.08	0.88	1679	632	17
Kruskall-Wallis test			p = 0.737	p = 0.985	p = 0.452	p < 0.001	p = 0.002	p = 0.403
Grading^a^								
1–2	59	(72.0)	1.04	1.04	0.82	1295	398	46
3	23	(28.0)	1.11	1.06	0.89	1501	948	0
Kruskall-Wallis test			p = 0.649	p = 0.485	p = 0.520	p = 0.112	p = 0.043	p = 0.135
HPV-driven oropharyngeal carcinoma								
No	12	(54.5)	0.85	1.02	0.89	1604	141	28
Yes	10	(45.5^b^)	1.20	1.10	0.82	1946	1277	15
Kruskall-Wallis test			p = 0.129	p = 0.199	p = 0.124	p = 0.396	p < 0.001	p = 1.000
Primary Treatment								
Surgery+/− (CT)RT	66	(64.4)	1.07	1.13	0.84	911	384	9
(CT)RT	35	(35.6)	1.03	1.04	0.89	1502	482	39
Kruskall-Wallis test			p = 0.272	p = 0.319	p = 0.456	p = 0.192	p = 0.449	p = 0.942

SM: Surrounding mucosa; PBMC: Peripheral blood monoclonal cells; (CT)RT: (chemo)radiotherapy.

^a^The figures do not add up to the total because of some missing values. ^b^The prevalence of HPV-driven oropharyngeal carcinoma is higher than has been previously reported and reflects the increasing trend in prevalence over the years.

### TERT levels in tumor, SM, and plasma

Median levels of *TERT* in tumor, SM, and plasma according to descriptive characteristics are reported in Table 1. *TERT* levels in tumor cells were significantly higher in females (*P* = 0.023) and in patients with advanced stage disease (*P* < 0.001) and neck metastases (*P* < 0.001). *TERT* levels in SM were significantly higher in patients with cancer of the hypopharynx/larynx (*P* = 0.013) and in those with advanced stage disease (*P* = 0.002). Among oropharyngeal cancers, patients with HPV-driven oropharyngeal cancer reported higher *TERT* levels in SM (*P* < 0.001). Circulating *TERT* levels in plasma were estimated in 97 cases; 42 cases, including 18 of those who had high tumor *TERT* levels, were negative for *TERT* mRNA. Overall, the analysis of *TERT* levels in plasma did not yield significant results (Table 1).

### Response to treatment

The correlation between telomere length and *TERT* levels with the response to treatment is summarized in Supplementary Table 2. In a multivariate analysis adjusted for gender, age and clinical variables it emerged that short telomeres in SM and high *TERT* levels in tumor tissue were significantly associated with worse treatment response (OR = 4.19, 95% CI: 1.01–17.47, *P* = 0.049 and OR = 6.26, 95% CI: 1.10–35.51, *P* = 0.038; respectively).

### Time-to-event analysis

During a median follow-up time of 20 months (interquartile range, 9–35 months), 27 patients (26.7%) had mucosal failure, 18 (17.8%) regional failures and 5 (5.0%) distant metastases. All patients with positive margins after surgery received adjuvant chemoradiation, and none developed mucosal failure. Overall, 39 patients (38.6%) experienced a PFS event and 32 patients (31.7%) died (24 deaths were attributable to cancer).

**Figure 2:**
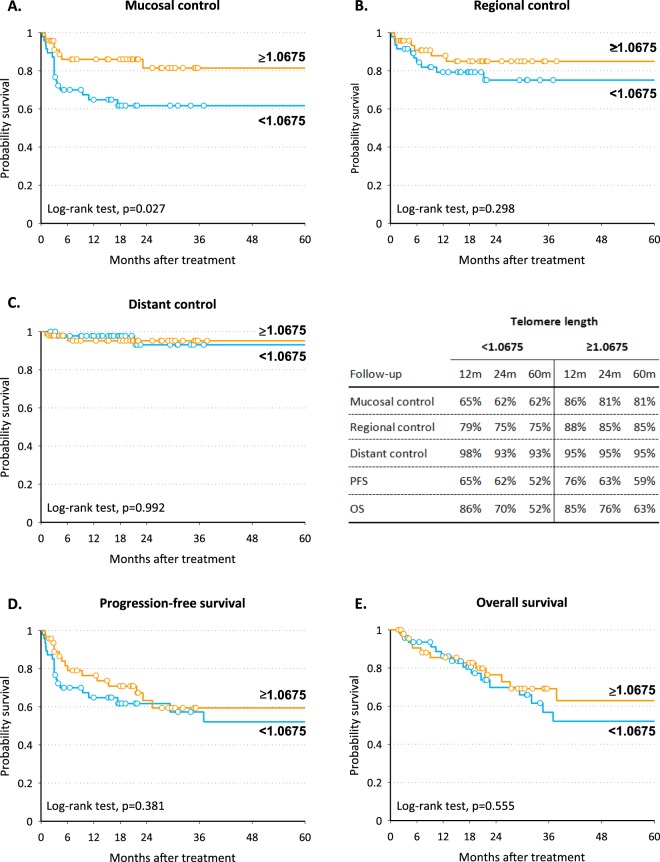
Kaplan-Meier estimates of mucosal control (**A**), regional control (**B**), distant control (**C**), progression-free survival (**D**) and overall survival (**E**) according to telomere length in surrounding mucosa.

**Figure 3:**
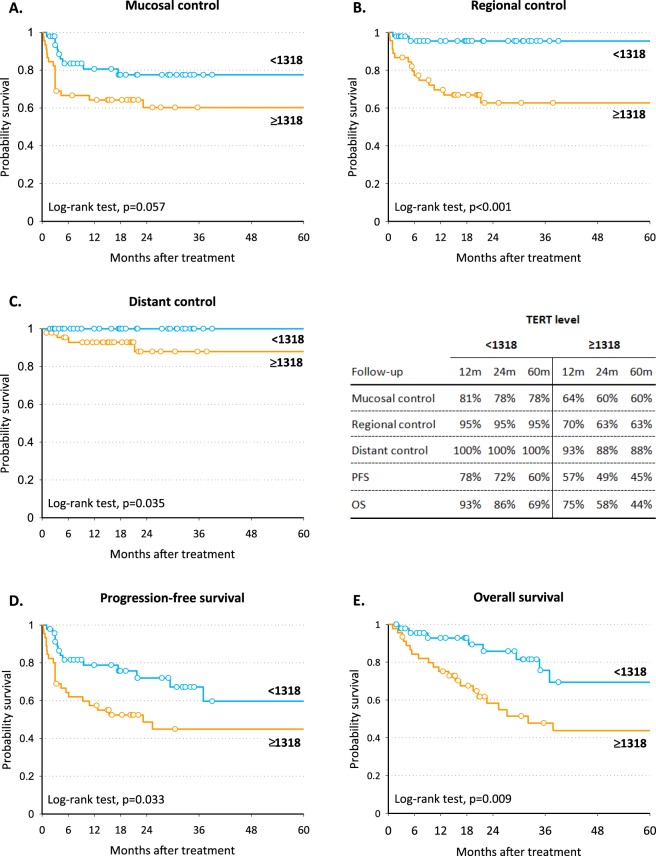
Kaplan-Meier estimates of mucosal control (**A**), regional control (**B**), distant control (**C**), progression-free survival (**D**) and overall survival (**E**) according to TERT level in tumor.

Short telomeres in SM were associated with worse mucosal control (5-year mucosal control = 62% vs 81% in patients with telomere length in SM < 1.0675 vs > 1.0675; *P* = 0.027; Fig. 2A). This association was confirmed by multivariate analysis (Table 2) after adjustment for clinical variables, with a HR for mucosal failure of 2.57 (95% CI: 1.03–6.42; *P* = 0.044). Telomere length in tumor tissue and PBMC was not associated with any of the clinical endpoints (Table 2). High *TERT* levels in tumor tissue were consistently associated with worse prognosis: 5-year OS was 69% in patients with *TERT* levels < 1318 and 44% in those with *TERT* levels > 1318 (*P* = 0.009; Fig. 3E). Corresponding figures for 5-year PFS were 60% and 45% (*P* = 0.033 Fig. 3D). This difference was likely due to worse regional control in patients with elevated *TERT* levels (95% vs 63%; *P* < 0.001 Fig. 3B). At multivariate analysis, high levels of *TERT* in tumor were significantly associated with a higher risk of regional failure (HR = 5.75, 95% CI: 1.16–28.49; *P* = 0.032), increased risk of progression (HR:2.12,; 95% CI: 1.00–4.47; *P* = 0.049) and death (HR:3.53, 95% CI: 1.47–8.52; *P* = 0.005) (Table 2). *TERT* levels in SM and plasma were not associated with any time-to-event variables (Table 2).

**Table 2:** Hazard ratio (HR) and corresponding 95% confidence interval (CI)a for mucosal failure, regional failure, distant failure, progression, and death according to strata of telomere length and telomerase reverse transcriptase (TERT) level.

	Patients	Mucosal failure^b^		Regional failure		Distant failure		Progression		Death	
		n	HR (95% CI)	n	HR (95% CI)	n	HR (95% CI)	n	HR (95% CI)	n	HR (95% CI)
Telomere length in tumor											
<1.0475	51	12	Ref	9	Ref	1	Ref	17	Ref	15	Ref
≥1.0475	49	15	1.84 (0.82–4.09)	9	1.43 (0.54–3.81)	4	—	22	1.84 (0.95–3.57)	17	1.49 (0.73–3.05)
			p = 0.138		p = 0.475				p = 0.07		p = 0.271
Telomere length in surrounding mucosa											
≥1.0675	47	7	Ref	6	Ref	2	Ref	15	Ref	12	Ref
<1.0675	48	18	2.57 (1.03–6.42)	11	1.62 (0.58–4.57)	3	1.22 (0.16–9.47)	22	1.45 (0.73–2.88)	18	1.28 (0.60–2.71)
			p = 0.044		p = 0.362		p = 0.849		p = 0.285		p = 0.525
Telomere length in PBMC											
≥0.878	30	5	Ref	4	Ref	2	—	7	Ref	5	Ref
<0.878	30	7	1.43 (0.41–5.04)	5	1.13 (0.24–5.29)	1	—	8	1.24 (0.42–3.70)	6	1.21 (0.33–4.46)
			p = 0.577		p = 0.878				p = 0.700		p = 0.779
TERT level in tumor											
<1318	46	9	Ref	2	Ref	0	Ref	13	Ref	8	Ref
≥1318	46	18	1.77 (0.69–4.54)	16	5.75 (1.16–28.49)	5	—	26	2.12 (1.00–4.47)	24	3.53 (1.47–8.52)
			p = 0.233		p = 0.032				p = 0.049		p = 0.005
TERT level in surrounding mucosa											
<441	44	11	Ref	6	Ref	1	Ref	17	Ref	13	Ref
≥441	43	14	1.03 (0.41–2.57)	11	1.46 (0.43–4.88)	4	1.86 (0.16–21.32)	20	0.91 (0.4–1.91)	17	1.25 (0.56–2.75)
			p = 0.948		p = 0.544		p = 0.618		p = 0.804		p = 0.587
TERT level in plasma											
0	40	11	Ref	5	Ref	2	Ref	15	Ref	13	Ref
≥1	53	13	0.91 (0.38–2.15)	10	1.87 (0.56–6.25)	2	1.87 (0.15–22.68)	20	1.01 (0.50–2.07)	15	0.61 (0.27–1.39)
			p = 0.824		p = 0.310		p = 0.623		p = 0.972		p = 0.239

^a^Estimated from Cox proportional hazard model, adjusting for gender, age, cancer site, stage, and surgery. ^b^Patients without complete response were considered recurred after 91days from end of treatment, if no earlier recurrence was reported.

PBMC = Peripheral blood mononuclear cells.

## Discussion

Data from this study confirm our previous observation, obtained from an independent series of patients^13^, that short telomeres in histologically normal SM are independently associated with a higher risk of mucosal failure. To investigate whether shortened telomeres in SM are the consequence of greater cell proliferation or are linked to the individual’s constitutive telomere length, we estimated telomere length in PBMC. Telomere length in PBMC is considered representative of an individual’s constitutive telomere length and correlates strongly with that in cells of different tissues, thus serving as a surrogate marker for other tissues^17^. In this series, while telomere length in PBMC did shorten with age thus showing normal telomeric dynamics, the lengths of telomeres in histologically normal mucosa surrounding UADT cancers did not correlate with patients’ ages or with telomere length in PBMC. Shortened telomeres in SM could therefore be a consequence of greater cell proliferation and/or environmental and lifestyle-related factors such as tobacco smoking and alcohol consumption. Local tumor recurrence at the primary site can be due to residual cancer cells or to the development of second field tumors. The majority (94%) of patients undergoing primary surgical treatment in this study had clear margins, and none of the four cases with close or involved margins suffered local mucosal failure. This suggests that second field tumors might play a crucial role in mucosal failure. The model of field cancerization has recently been reviewed in molecular terms^18^. According to the model, precancerous fields characterized by genetic alterations due to carcinogen exposure can precede the emergence of synchronous or more frequently metachronous multiple cancer lesions. Part of this at-risk mucosa remains after the primary tumor has been treated and is the site of origin of subsequent lesions^18,19^. A more recent investigation found that the majority of the genomic changes, i.e. copy number alterations and point mutations, driving oral carcinogenesis take place in the pre-cancerous condition through gradual casual accumulation rather than as a consequence of a dramatic single event^20^.

Interestingly, patients exposed to tobacco and alcohol had shorter telomeres in histologically normal SM with the association being statistically significant in ever drinkers. Overall, the comparison of telomere length in adjacent mucosa and in PBMC in respect to age indicates that the phenotypically normal mucosa surrounding the tumor constitutes an acquired telomere-shortened epithelial field prone to genetic instability. This hypothesis is supported by findings of previous investigations that observed the presence of *TP53* mutations in the field of macroscopically normal mucosa surrounding HNSCC in more than one-third of patients^21^ and the generation, due to telomere dysfunction in *TP53* mutant mice, of non-reciprocal translocations initiating the neoplastic process and accelerating carcinogenesis^22^.

Furthermore, we found that short telomeres in SM are associated with a worse response to multimodal therapy, including disease progression during treatment. According to some studies^18,19^, the cancer cells of the primary tumor and the genetically unstable cells of the SM belong to a common pre-neoplastic field. Most patients treated with radical surgical resection of the tumor show genetically altered margins upon molecular analysis, and appear to be at high risk of disease progression since the genetically altered mucosa surrounding the primary tumor progressively transforms. Other theories argue that disease progression is due to the acquired migratory capacity of the neoplastic cells through the submucosa or to their excretion in the lumen of a hollow organ (e.g. UADT) with subsequent regrowth in nearby locations^23,24^. Confirmation of these results and determination of their clinical implications might lead to more aggressive therapeutic strategies, more accurate evaluation of the disease response under therapy, and a stricter and more personalized follow-up regime in patients with shorter telomeres in SM. Although there is evidence that telomere function can influence radiosensitivity^15,16^ it was not possible to assess this in our clinical cohort because most of patients received (chemo)radiotherapy as upfront treatment, or as adjuvant therapy following surgical treatment and the small number of patients treated by surgery alone did not allow to explore this hypothesis.

Tumors harboring higher *TERT* levels were more frequently diagnosed in advanced stage and associated with the involvement of the regional lymph nodes. Moreover, risk of regional failure, progression, and death were independently associated with high levels of *TERT* thus confirming previous data showing that *TERT* levels in cancer tissue increase with the aggressiveness of the disease^10,13,25,26^. This association may be attributable to telomerase’s non-canonical functions which are implicated in regulating several cancer hallmarks including cell proliferation, resistance to apoptosis, invasion, and metastasis by interaction of TERT with important cancer-related signaling cascades, such as Wnt/β-catenin and NF-kB pathways^10,27^. The activation of NF-kB signaling pathway by TERT stimulates the epithelial-to-mesenchymal transition that provides cancer cells with a more migratory mesenchymal phenotype^12^. High tumor *TERT* levels also correlate with a worse response to treatment, and it has been demonstrated that TERT confers resistance to different apoptotic stimuli including treatment with chemotherapeutic agents and radiation^28–30^. Accordingly, TERT inhibition, through activation of DNA damage response non related to telomere shortening, sensitizes cells to the pro-apoptotic effect of chemotherapeutic drugs^31^. Thus, it is conceivable to attribute the negative predictive value of *TERT* levels to its previously discussed extra-telomeric properties. Furthermore, patients with HPV-driven oropharyngeal cancer had higher levels of *TERT* in SM. This observation may be related to the topographic restriction of HPV-driven HNSCC to the lymphoepithelial tissue of the palatine tonsils and base of the tongue, potentially characterized by lymphocytes overexpressing *TERT*^13,32,33^, that with high probability could have been collected when the sampling of the mucosa surrounding the oropharyngeal carcinomas was performed.

Circulating cell-free *TERT* mRNA levels have been found to be higher in cancer patients with respect to controls, as well as independent markers of tumor response, and prognostic of disease progression in cancers frequently presenting with high tumor mass, e.g. gastric and colorectal cancer^25,34,35^. In the present study, we failed to find an association between plasma *TERT* levels and outcome variables. This negative result may be due to the low tumor volume and the greater propensity of HNSCC to spread along lymphatic channels rather than hematogenous routes^36^. In a substantial fraction of cases it was not possible to detect specific *TERT* mRNA in the plasma. It has been proposed that plasma circulating *TERT* levels could derive from activated lymphocytes and therefore they may be indicative of the induction of tumor immune responses^37^. Further studies are needed to clarify the significance and the role of circulating *TERT* levels in HNSCC.

In conclusion, high levels of *TERT* transcripts in cancer cells represent a reliable prognostic marker for identifying HNSCC patients with worse response to treatment and risk of progression. The different behavior of telomere length in adjacent mucosa and PBMC in respect to age suggests that in a subset of patients the phenotypically normal mucosa surrounding the tumor constitutes an acquired telomere-shortened epithelial field prone to genetic instability. These observations may lead to new perspectives in risk stratification, treatment, and surveillance strategies in patients with HNSCC.

## Methods

### Patients

In total, 101 consecutive newly diagnosed patients with histologically confirmed HNSCC (larynx, oral cavity, oropharynx and hypopharynx) were included in this prospective cohort study from 2012 to 2017. Due to differing epidemiology, etiology, histology and management, patients with nasopharyngeal carcinoma were excluded. Irrespective of HPV status, a multidisciplinary team decided on treatment planning according to TNM staging. Most T1 and T2 HNSCCs were treated with function-preserving surgery or definitive radiotherapy. Most patients with T3 and T4 tumors underwent radical surgery followed by post-operative (chemo)-radiotherapy depending on the presence of adverse features, or upfront chemoradiation. The local institutional review board approved the study protocol (“Comitato Etico Sperimentazione Clinica per le Province di Treviso e Belluno”, Italy; ethic vote: 346/AULSS9) and all patients gave their informed consent. We confirm that all experiments were performed in accordance with relevant guidelines and regulations. Along with the blood sample, two solid tissue samples were collected from each patient before treatment, one from a non-necrotic area of the carcinoma, and the other from SM. A UADT fiberoptic evaluation as well as computed tomography (CT) and/or magnetic resonance imaging (MRI) of the primary tumor and the neck were carried out 8–12 weeks after treatment to assess tumor response. The routine follow-up program consisted of locoregional examination including UADT endoscopy at 2-month intervals during the first year, 3-month intervals in the second year, 4-month intervals between the third and fifth year, and every 6 months thereafter.

### Tissue samples

Tissues samples from both tumor and SM were obtained before treatment. Surgeons were also requested to biopsy uninvolved mucosa at around 4 cm from the tumor margins depending on anatomical location. Precautions were taken to not contaminate the SM with tumor samples by changing surgical blades each time before cutting tissue. SM tissue was available for 96 patients. Both samples were snap-frozen in liquid nitrogen and stored at −80 °C until analysis. Cryostat sections, 6 microns thick, from each tissue sample were prepared using a 1720 Digital cryostat (Leitz, Germany). One section of each sample was stained with haematoxylin-eosin for histopathology. SM was histologically assessed and, in all cases, no histopathological alterations were found. For each tumor and SM sample, 5 or 6 cryostat sections were collected into two different 2 mL eppendorf tubes and stored at −80 °C and −20 °C respectively, for RNA and DNA extraction. DNA was extracted by the standard phenol/chloroform method, while RNA was extracted with Trizol reagent (Life Technologies, Carlsbad, CA, USA) and reverse-transcribed into cDNA using the SuperScript TM III RNase reverse trancriptase assay (Thermo Fisher Scientific, Waltham, MA, USA), according to the manufacturer’s instructions.

### Peripheral blood samples

Plasma and PBMC samples were obtained, before treatment, from 6 mL of peripheral blood by Ficoll Paque (GE Healthcare, Chicago, IL, USA) protocol, and stored at −80 °C and −20 °C respectively, until use. DNA for telomere length analysis was extracted from PBMC by the standard phenol/chloroform method, while the circulating mRNA for the quantification of *TERT* transcripts was extracted from plasma samples as previously described^34^, with the only modification that in this series of samples we started from 1 ml instead of 500 μL of plasma and all reagent quantities were adjusted accordingly. RNA was reverse transcribed into cDNA using the SuperScript TM III RNase reverse trancriptase assay (Thermo Fisher Scientific) in a final volume of 80 μL, according to the manufacturer’s instructions.

### Quantification of TERT transcripts in tissue and plasma samples

The level of *TERT* transcripts was quantified by real-time PCR, as previously described^25^ with some modifications. In particular, a new primer pair was designed in order to reduce the length of the amplified product, thus improving detection of the cDNA target sequence^38,39^. Primers AT1 (5′-CGGAAGAGTGTCTGGAGCAA-3′) and AT2b (5′-CGCAGCTGCACCCTCTTCA-3′) were designed on exon 3 and 4 respectively; they bind to nucleotide sequences located upstream of the RT motif 1 on the *TERT* gene allowing amplification of all *TERT* transcripts producing an amplified product of 68 bp^34^. Values of *TERT* were normalized for 10^3^ copies of *HPRT1*. For plasma samples, levels of *TERT* mRNA were also estimated per mL using the conversion factor x 8 as previously described^34^.

### Telomere length measurement

Relative telomere length was determined on DNA extracted from tissues and PBMC by multiplex quantitative Real-time PCR, as previously described^40,41^, with minor modifications. In particular, each PCR reaction was performed in a final volume of 25 μL, containing 5 μL sample (10 ng DNA) and 20 μL master-mix ready-to-use 1X Light Cycler 480 SYBR Green I (Roche Life Science, Penzberg, BY, Germany), containing 900 nmol/L of each primer. Sequences of telomere and albumin primers are detailed in a previous study^40^. The thermal cycling profile was 15 min at 95 °C, two cycles of 15 s at 94 °C, 15 s at 51 °C, followed by 40 cycles of 15 s at 94 °C, 10 s at 62 °C, 15 s at 74 °C, 10 s at 84 °C, 15 s at 89 °C, with signal acquisition at the end of both the 74 °C and 89 °C steps. After cycling, a melting curve program was run starting with a 95 °C incubation for 1 minute, followed by continuous acquisitions every 0.2 °C for 45 °C to 95 °C (ramping at 0.11 °C/s). A standard curve was generated at each PCR run, consisting of DNA from the RAJI cell line, serially diluted from 10 to 0.41 ng/µl^42^. All DNA samples and reference samples were run in triplicate. LightCycler raw text files were converted using the LC480Conversion free software (http://www.hartfaalcentrum.nl/index.php?main=files&fileName=LC480Conversion.zip&description=LC480Conversion:%20conversion%20of%20raw%20data%20from%20LC480&sub=LC480Conversion), and the converted data were analysed using LinRegPCR free software^43^. Telomere length values were calculated as telomere/single-copy gene (T/S) ratio, as previously described^42^.

### Statistical analysis

Telomere length in tumor, SM, PBMC according to age groups were displayed by beeswarm plots. The association between telomere length and age was tested through the analysis of variance (ANOVA) with contrasts for linear trend. Differences in telomere length and *TERT* level according to socio-demographic and clinical characteristics were tested with the Kruskall-Wallis test.

The association between telomere lengths, *TERT* levels, and treatment response was evaluated by calculating the odds ratio (OR) of partial response or disease progression versus complete response through unconditional logistic regression model. OR and 95% corresponding confidence intervals (CI) were adjusted for gender, and potential clinical confounders (namely, cancer site, stage, and surgery).

The impact of telomere length and *TERT* levels on survival outcomes was evaluated through survival analysis, comparing patients with telomere length and *TERT* levels under (low) or above (high) tissue-specific median values. For each patient, person-time at risk was computed from the date of last treatment to the event date or the date of last follow-up, whichever came first. Event was defined as cancer reappearance at the UADT site for mucosal control; neck lymph node failure for regional control; distant metastasis for distant control; mucosal, regional or distant recurrence or death for progression-free survival; death for overall survival. The Kaplan-Meier method was used to generate crude survival curves and the log-rank test was used to assess the heterogeneity in time to event in strata of selected covariates^44^, censoring follow-up at 5 years. Hazard ratios (HR) and the corresponding 95% CI were calculated using Cox proportional hazards models^44^, adjusting for gender, age, and potential confounders (namely cancer site, stage, and surgical treatment).

## Supplementary information

Supplementary tables 1 & 2

## Data Availability

The datasets generated during and/or analyzed during the current study are available from the corresponding author on reasonable request.
