# Supplementary material for: Predictive and prognostic significance of telomerase levels/telomere length in tissues and peripheral blood in head and neck squamous cell carcinoma
Source: Sci Rep. 2019 Nov 26;9:17572. doi: 10.1038/s41598-019-54028-x (PMC6879742; doi:10.1038/s41598-019-54028-x)
Supplement: Supplementary file 1 — Supplementary tables 1 & 2 [file 41598_2019_54028_MOESM1_ESM.pdf]

Title

Predictive and prognostic significance of telomerase levels/telomere length in tissues and peripheral blood in head and neck squamous cell carcinoma

Authors

Paolo Boscolo-Rizzo, Enrica Rampazzo, Jerry Polesel, Silvia Giunco, Anna Menegaldo, Monica Mantovani, Marco Stellin, Luigia Bandolin, Giacomo Spinato, Annarosa Del Mistro, Daniele Borsetto, Jonathan Fussey, Giancarlo Tirelli, Maria Cristina Da Mosto, Anita De Rossi

Supplementary table 1. Distribution of 101 patients with head and neck cancer according to patients/tumor characteristics, by anatomical subsite.

	All		Anatomical subsite					
			Oral cavity		Oropharynx		Hypopharynx/ Larynx	
	n	(%)	n	(%)	n	(%)	n	(%)
Gender								
Male	74	(73.3)	18	(66.7)	11	(50.0)	45	(86.5)
Female	27	(26.7)	9	(33.3)	11	(50.0)	7	(13.5)
					p=0.003			
Age (years)								
<60	30	(29.7)	9	(33.3)	7	(31.8)	14	(26.9)
60-69	37	(36.3)	7	(25.9)	9	(40.9)	21	(40.4)
≥70	34	(33.3)	11	(40.7)	6	(27.3)	17	(32.7)
					p=0.713			
Smoking habits								
Never	27	(26.7)	9	(33.3)	7	(31.8)	11	(21.2)
Ever	74	(73.3)	18	(66.7)	15	(68.2)	41	(78.8)
					p=0.424			
Drinking habits								
Never	41	(40.6)	12	(44.4)	14	(63.6)	15	(28.9)
Ever	60	(59.4)	15	(55.6)	8	(36.4)	37	(71.1)
					p=0.018			
cT								
T1-T2	52	(51.5)	20	(74.1)	7	(31.8)	25	(48.1)
T3-T4	49	(48.5)	7	(25.9)	15	(68.2)	27	(51.9)
					p<0.001			
cN								
Negative	50	(49.5)	19	(70.4)	3	(13.6)	28	(53.9)
Positive	51	(50.5)	8	(29.6)	19	(86.4)	24	(46.1)
					p=0.003			
Stage								
I-II	36	(35.6)	15	(55.6)	2	(9.1)	19	(36.5)
III-IV	65	(64.4)	12	(44.4)	20	(90.9)	33	(63.5)
					p=0.003			
Treatment								
Upfront surgery	66	(65.3)	24	(88.9)	10	(45.4)	32	(61.5)
Upfront RCT	35	(34.7)	3	(11.1)	12	(54.6)	20	(38.5)
					p=0.005			

Supplementary Table 2. Odds ratio (OR) and corresponding 95% confidence interval (CI) for treatment response (partial response/progression disease vs. complete response) according to telomere length and TERT level.

	PR/PD		CR		Univariate[a]		Multivariate[b]	
	n	(%)	n	(%)	OR	(95% CI)	OR	(95% CI)
Telomere length in tumor								
<1.0475	8	(44.4)	42	(51.9)	Ref		Ref	
≥1.0475	12	(55.6)	39	(48.2)	1.25	(0.44-3.59) p=0.673	1.36	(0.37-5.01) p=0.649
Telomere length in surrounding mucosa								
≥1.0675	4	(25.0)	42	(53.9)	Ref		Ref	
<1.0675	12	(75.0)	36	(46.2)	4.18	(1.17-14.88) p=0.027	4.19	(1.01-17.47) p=0.049
Telomere length in PBMC								
≥0.878	3	(33.3)	27	(52.9)	Ref		Ref	
<0.878	6	(66.7)	24	(47.1)	2.31	(0.51-10.58) p=0.280	4.97	(0.64-38.44) p=0.125
TERT level in tumor								
<1318	3	(16.7)	42	(57.5)	Ref		Ref	
≥1318	15	(83.3)	31	(42.5)	7.61	(1.87-31.00) p=0.005	6.26	(1.10-35.51) p=0.038
TERT level in surrounding mucosa								
<441	5	(31.3)	38	(54.3)	Ref		Ref	
≥441	11	(68.8)	32	(45.7)	2.59	(0.81-8.32) p=0.109	1.33	(0.29-6.17) p=0.714
TERT level in plasma								
0	6	(40.0)	33	(42.9)	Ref		Ref	
≥1	9	(60.0)	44	(57.1)	1.35	(0.42-4.36) p=0.615	0.88	(0.17-4.60) p=0.881

[a]Estimated from unconditional logistic regression model, adjusting for gender and age. [b]Further adjusted for cancer site, stage, and surgery.

PBMC= Peripheral blood mononuclear cells
